# Leader–Member Exchange, Work Engagement, and Psychological Withdrawal Behavior: The Mediating Role of Psychological Empowerment

**DOI:** 10.3389/fpsyg.2020.00423

**Published:** 2020-03-31

**Authors:** Arun Aggarwal, Pawan Kumar Chand, Deepika Jhamb, Amit Mittal

**Affiliations:** Chitkara Business School, Chitkara University, Punjab, India

**Keywords:** leader–member exchange, psychological empowerment, work engagement, psychological withdrawal behavior, structural equation modeling, research and development

## Abstract

Perceptions of psychological empowerment play a vital role in the way an individual perceives things at the workplace. In spite of this, there is scant research on the antecedents and consequences of psychological empowerment. This study is an attempt to fill this gap by analyzing the mediating role of psychological empowerment on the relationship between its antecedents (leader–member exchange) and its consequences (work engagement and psychological withdrawal behavior). Data were collected from 454 employees working in the Research and Development (R&D) departments of the information technology (IT) and pharmaceutical sectors operating in India. Results suggest that employees who have a high-quality relationship with their leader have high psychological empowerment, they are highly engaged at work, and their psychological withdrawal behavior is also low. In addition to this, high levels of psychological empowerment have a positive impact on their engagement toward work, which further leads to a low psychological withdrawal behavior. The theoretical and practical implications of these results are discussed.

## Introduction

In recent years, leader–member exchange (LMX) has gained a lot of attention from researchers because of its consequences on employees’ work performance (Epitropaki et al., 2016; Schwepker, 2017; Siyal and Peng, 2018). LMX is one of the most prominent theories that deal with the dual relationship between a leader and the subordinates (Graen and Wakabayashi, 1994; Brower et al., 2000; Pellegrini et al., 2010). The underlying premise of this theory is that leaders develop a diverse relationship with their subordinates ranging from low (out-group) to high (in-group) quality (Graen and Wakabayashi, 1994; Green et al., 1996; Brower et al., 2009; Dulebohn et al., 2012). A high-quality LMX leads to a higher level of information exchange, trust, competence, commitment, role clarity, greater job satisfaction, and lower job stress (Wang and Yi, 2011; Chernyak-Hai and Tziner, 2014; Martin et al., 2016; Lebrón et al., 2018). On the other hand, a low-quality LMX leads to a low level of interaction, limited support, formal relations, counterproductive behavior, psychological withdrawal behavior, employee turnover, lower level of job satisfaction, and higher job stress (Harris et al., 2005; Wang and Yi, 2011; Lebrón et al., 2018).

According to LMX, leaders evaluate their subordinates based on multiple parameters such as agreeableness, competence, conscientiousness, locus of control, neuroticism, extraversion, openness, and positive, and negative affectivity (Erdogan and Liden, 2002; Dulebohn et al., 2012; Clarke, 2016; Inanc, 2018). On the other hand, leaders are judged on the basis of contingent reward behavior, transformational leadership, supervisor’s expectation of followers, agreeableness, and extraversion (Judge and Piccolo, 2004; Anand et al., 2011; Bedi et al., 2016). While looking at the importance of the dyadic relationship between employee and employer, the present study is an attempt to identify the mediating impact of psychological empowerment on the relationship between LMX and its outcome of work engagement and psychological withdrawal behavior of employees working in the Research and Development (R&D) departments of the information technology (IT) and pharmaceutical sectors.

Psychological empowerment is one of the significant consequences of high-quality LMX. Psychological empowerment is defined as an “intrinsic task motivation reflecting a sense of self-control in relation to one’s work and an active involvement with one’s work role” (Seibert et al., 2011, p. 981). It is an important component of workplace empowerment constituting intrinsic task motivation or employee rewards underlying the strengthened working conditions (Aggarwal et al., 2018a; Laschinger et al., 2009). Employees’ empowerment in any organization further results in allocating meaningful work, self-efficacy, self-determination, and competence, which are the major elements of psychological empowerment (Aryee and Chen, 2006; Harris et al., 2009; Aggarwal et al., 2019b). These elements reflect employees’ orientation toward their jobs and are associated with positive results. From the empirical evidence, it has been found that both LMX and psychological empowerment are positively related to organizational behavior (Schermuly and Meyer, 2016; Hu et al., 2018). The other important consequence of high-quality LMX is work engagement (Radstaak and Hennes, 2017; Lebrón et al., 2018; Kapil and Rastogi, 2019). Macey et al. (2011, p. 5) defined work engagement as a “psychic kick of immersion, striving, absorption, focus, and involvement.” According to Breevaart et al. (2015, p. 755), “Engaged employees have high levels of energy, are enthusiastic about, inspired by, and proud of their work, and feel like time flies when they are working.” It involves investing “hands, head, and heart” inactive, full work performance (Agarwal et al., 2012). There is a higher tendency that the employees who experience high-quality relationships at their workplace feel psychologically safe (Halbesleben, 2010; Gruman and Saks, 2011). The sense of psychological safety further enhances employees’ work engagement (Lonsdale, 2016; Garg and Dhar, 2017). In this study, the authors claim that high-quality LMX is positively related to work engagement.

Despite being a heavily researched area, there are very few efforts by previous researchers to identify the relationship between high-quality LMX and psychological withdrawal behavior (Martin et al., 2016; Lebrón et al., 2018). Lehman and Simpson (1992) described psychological withdrawal behavior as “an aggregate of neglect behaviors at work and has been reported to be negatively related to performance.” Withdrawal behaviors refer to a “set of attitudes and behaviors seen in employees whose job performance has deteriorated” (Shapira-Lishchinsky and Even-Zohar, 2011, p. 429). A high-quality relationship enhances a sense of freedom and delegates power from superiors to their subordinates, which ultimately helps in reducing employees’ withdrawal behavior (Dollard and Idris, 2017; Landells and Albrecht, 2017). Therefore, the authors attempt to expand this line of research by claiming that high-quality LMX leads to low psychological withdrawal behavior.

The purpose of this study is to add new knowledge to the existing literature of organizational behavior by examining how the quality of LMX affects psychological empowerment which further affects the employees’ level of engagement toward the organization and their psychological withdrawal behavior. The present study is the first of its kind to explore the LMX, psychological empowerment, work engagement, and psychological withdrawal behavior altogether.

## Theoretical Framework

There is an increasing trend among organizational researchers to study the effect of LMX on various work-related consequences (Dulebohn et al., 2012; Schermuly and Meyer, 2016). According to Graen and Uhl-Bien (1995), the LMX theory is a relationship-based approach to leadership in which leaders develop varying relationships with their followers based on their exchanges and interactions. A leader develops either high or low dyadic relationships with his/her subordinates (Tabak and Hendy, 2016; Chernyak-Hai and Rabenu, 2018). The basis of LMX is that “dyadic relationships and work roles are developed and negotiated over time through a series of exchanges between the leader and member” (Bauer and Green, 1996, p. 1538). These subdimensions of LMX are correlated to such an extent that “they can be tapped into with the single measure of LMX” (Graen and Uhl-Bien, 1995, p. 237). Hence, in the present research, we consider LMX as unidimensional rather than multidimensional (Bernerth et al., 2007; Schermuly and Meyer, 2016). High LMX indicates mutual respect, likings between both the parties, and positive interaction with the followers, which go beyond the formal job description (Nahrgang et al., 2009). In contrast, subordinates who perform only in accordance with the prescribed employment contract are characterized as “out-group” with limited reciprocal trust and support and few rewards from their supervisors (Deluga, 1998). According to relative deprivation theory, whenever followers face discrepancies under low LMX, there are two possibilities. Firstly, look ahead for self-improvement comparing the others, and secondly, follow the actions of counterproductive work behavior such as psychological withdrawal behavior (Crosby, 1976; Bolino and Turnley, 2009; Shkoler and Tziner, 2017; Lebrón et al., 2018). Employees under low LMX encounter a low scope of psychological empowerment and low job satisfaction. The three moderators in deprivation are first, limited interaction of employee for LMX support and development; second, follower self-efficacy; and third, assessment of leader and follower relationship by the leader.

## Hypotheses Development

### Leader–Member Exchange and Psychological Empowerment

LMX emerged as a positive organizational factor and has drawn the attention of the researchers to understand the supervisors’ and subordinates’ relationship (Cropanzano and Mitchell, 2005). High LMX supports the organizational culture by building trust, sharing of information, resources, rewards, loyalty, and openness (Erdogan et al., 2006; Asgari et al., 2008; Chernyak-Hai and Rabenu, 2018). Employees under high LMX express themselves better in the organization, have a greater sense to work context and a positive attitude to accept the work challenges, and show innovativeness. Employees who perceive their relationship with the leader as high perform better than employees who have a low dyadic relationship with the leader and have a strong ability to adapt to changes (Liden et al., 2000; Chen and Klimoski, 2003; Carson and King, 2005). Previous research has shown that high LMX leads to high psychological empowerment among the employees (Hill et al., 2014; Wang et al., 2016; Newman et al., 2017; Hu et al., 2018). This leads to the first hypothesis.

H1: Leader–member exchange positively impacts psychological empowerment

### Leader–Member Exchange and Work Engagement

LMX enhances the work engagement of the employees by the characteristics of vigor, dedication, and absorption (Schaufeli et al., 2006; Halbesleben, 2010; Christian et al., 2011; Sharoni et al., 2015; Rabenu et al., 2019). There is an enhancement in the level of work engagement and job performance when employees frequently interact with their supportive leader, which further leads to a better job design, organizational culture, and resource distribution (Attridge, 2009; Bakker and Xanthopoulou, 2009). A high-quality dyadic relationship makes the supervisor look ahead for numerous interactions with subordinates, get attached emotionally with them, and provide them psychological empowerment, which further leads to a high work engagement (Tabak and Hendy, 2016). Work engagement is a motivational concept because it makes the employees struggle hard for challenging goals and gives them the inspiration to succeed in them (Leiter and Bakker, 2010). Prior research findings have shown a positive relationship between LMX and work engagement (Agarwal et al., 2012; Runhaar et al., 2013; Burch and Guarana, 2014; Matta et al., 2015; Garg and Dhar, 2017).

H2: Leader–member exchange positively impacts work engagement

### Leader–Member Exchange and Psychological Withdrawal Behavior

There is a scarcity of research on the relationship between LMX and psychological withdrawal behavior (Martin et al., 2016; Lebrón et al., 2018). Despite the fact that the role of LMX is very vital in controlling psychological withdrawal behavior. Low LMX leads to poor interaction between leaders and followers, poor leadership support, and a high level of stress among employees, frustration, violations, and negative affectivity (Griffeth et al., 2000; Glasø and Einarsen, 2006). Employees under withdrawal behavior exhibit low morale, feel stressed, and realize the work pressure negatively (Shapira-Lishchinsky and Rosenblatt, 2009). Psychological withdrawal behaviors can be traced as willful lateness (Blau et al., 2004), intent to leave, and absenteeism (Koslowsky, 2009; Biron and Bamberger, 2012). Psychological withdrawal behavior describes the employees’ behavior and attitudes responsible for the low level of job performance at the workplace (Johns, 1997; Shaw et al., 2005; Kaplan et al., 2009; Shapira-Lishchinsky and Rosenblatt, 2010). A low level of the social exchange relationship between leader and followers lowers down the employees’ performance, commitment, and the job satisfaction level of the employees at the workplace (Rhoades and Eisenberger, 2002).

H3: Leader–member exchange negatively impacts psychological withdrawal behavior

### Psychological Empowerment and Work Engagement

Psychological empowerment comprises four elements, namely, meaning, competence, self-determinations, and impact (Sparrowe, 1994; Kirkman and Rosen, 1999; Siegall and Gardner, 2000). Previous literature has manifested that psychological empowerment has a positive impact on work engagement of the employees (Paré and Tremblay, 2007; Bakker and Leiter, 2010; Stander and Rothmann, 2010; Seibert et al., 2011; Wang and Liu, 2015; Al-Maamari et al., 2017). Alzyoud et al. (2015) state that higher work engagement enhances the commitment and job satisfaction among the employees and reduces employee absenteeism at the workplace. Job Demands–Resources model also states that employees are found to be more engaged at the work that offered empowerment in psychological conditions such as organization culture, job enrichment, and opportunity to work under supportive leadership (Bakker et al., 2014). Therefore, it was hypothesized that:

H4: Psychological empowerment positively impacts work engagement

### Psychological Empowerment and Psychological Withdrawal Behavior

Under psychological withdrawal behavior, employees tend to depart themselves from their respective workplace and they have a negative attitude toward their work. These negative attitudes include turnover intentions, intentional absenteeism, and lateness at their workplace (Johns, 1997; Shapira-Lishchinsky and Rosenblatt, 2010; Shapira-Lishchinsky and Tsemach, 2014). Employees under psychological withdrawal behavior influence other employees to contribute lesser efforts at the workplace, and such employees were also found frequently switching jobs (Hoendervanger et al., 2019). Therefore, it is important to understand the factors that affect the employees’ psychological withdrawal behavior in the organizational context. One such important factor that affects the psychological withdrawal behavior is psychological empowerment (Dewettinck and van Ameijde, 2011; Shapira-Lishchinsky and Tsemach, 2014; Bester et al., 2015). When employees are able to positively impact the working conditions at their workplace (“impact” sub-factor of psychological empowerment), when employees are competent to perform their respective jobs (“competence” sub-factor of psychological empowerment), when employees are free in taking their own decisions (“self-determination” sub-factor of psychological empowerment), and when employees perceive their job as meaningful (“meaning” sub-factor of psychological empowerment), in that scenario, it is more likely that their attachment toward the workplace and work will be high (Shapira-Lishchinsky and Tsemach, 2014). Therefore, when the individual is psychologically empowered, he/she shows high job satisfaction and negligible psychological withdrawal behavior (Fook et al., 2011). Therefore, it was hypothesized that:

H5: Psychological empowerment negatively impacts psychological withdrawal behavior

### Work Engagement and Psychological Withdrawal Behavior

Psychological withdrawal behavior may prone the employee to show laziness or lack of intense thinking on the job (Pinder, 2008). The disengaged employee with withdrawal behavior can cause loss to the organization in billions of rupees every year (Rosch, 2001; Berry et al., 2012). According to Gallup’s survey 2011–2012 (Crabtree, 2013), the global percentage of engaged employees in the organization is found to be at 13%, which is very alarming. Previous research shows a significant relationship between work engagement and psychological withdrawal behavior (Malinen et al., 2013; Shusha, 2013; Huang et al., 2016; De Simone et al., 2018). Therefore, it is essential to understand the relationship between work engagement and psychological withdrawal behavior in the workplace.

H6: Work engagement negatively impacts psychological withdrawal behavior

### Psychological Empowerment as a Mediator

Recent research work in organizational behavior has focused on examining the mediating role of psychological empowerment in different workplace relations (Schermuly and Meyer, 2016; Hu et al., 2018). Prior research has shown that the quality of the relationship between leader and follower affects the level of psychological empowerment perceived by the followers (Harris et al., 2009; Hill et al., 2014). Leader’s ease of availability and his/her supportive behavior helps in improving the psychological empowerment of the employees (Hu et al., 2018). Furthermore, this psychological empowerment leads to various organizational consequences such as high work engagement (Wang and Liu, 2015; Al-Maamari et al., 2017) and low psychological withdrawal behavior (Colquitt et al., 2014; Lorinkova and Perry, 2017). The role of the social exchange relationship between leader and subordinate was found imperative to enhance the work engagement (Carasco-Saul et al., 2015; Galperin et al., 2017). Literature states that few researchers explore the significant positive indirect relationship of LMX and work engagement in the presence of mediating variable employee empowerment (De Villiers and Stander, 2011; Mendes and Stander, 2011). Empowering the employee improves the job outcomes and work engagement because the leader shares the powers with the employees (Vecchio et al., 2010; Tuckey et al., 2012; Wang et al., 2016) such as delegation of authority and giving them opportunities to participate in the decision-making process (Zhang and Bartol, 2010). Researchers also found the positive significant relationship between LMX and employee empowerment and explained that employee empowerment can be a mediator between LMX and job outcome variables such as work engagement, employee satisfaction, job performance, and innovative behavior (Dulebohn et al., 2012; Zhou et al., 2012; Schermuly and Meyer, 2016; Ciftci, 2019). Hence, it becomes important to examine the relationship between LMX and work engagement of employees in the presence of psychological empowerment as mediator.

H7a: Psychological empowerment mediates the relationship between leader–member exchange and work engagement

Fong and Snape (2015) explained that the psychological empowerment of employees affects the individuals’ attitudes and behavior in an organization. Previous research shows the positive effect of LMX on psychological empowerment, intrinsic motivation, and job performance of employees (Arnolds and Boshoff, 2000; Avolio et al., 2004; Zhang and Bartol, 2010). Further, some researchers also found that the relationship between effective leadership, LMX, absenteeism, and emotional exhaustion is mediated by psychological empowerment (Frooman et al., 2012; Kim and Beehr, 2018). In addition to this, employee withdrawal behavior is linked directly to high investment costs in the organization (Berry et al., 2012; Hancock et al., 2013). Low level of LMX can be a responsible factor for a low level of social interactions between the leader and the followers, which further leads to a lower level of interest among employees and turn them to show the withdrawal behavior such as absenteeism and turnover intention (Bolino and Turnley, 2009; Portoghese et al., 2015). Hence, it is necessary to understand the indirect effect of LMX on employee withdrawal behavior in the presence of mediator as employee empowerment.

H7b: Psychological empowerment mediates the relationship between leader–member exchange and psychological withdrawal behavior

## Methods

### Participants and Procedures

The data were collected from employees engaged in the R&D department, working in the IT sector and pharmaceutical sector operating in different cities in India, namely, Chandigarh, Delhi (and its extended suburbs Gurugram, Noida, and Ghaziabad), and Bangaluru. The participants were assured about the confidentiality of their data and data were taken from those employees who were interested in filling the questionnaire. No incentive was given to any employee for filling the questionnaire. Data were collected by administering a structured questionnaire to 1,163 employees through off-line mode out of which 454 usable questionnaires were received for final analysis with a response rate of 39.03%. The rest of the 709 questionnaires were not included in the final analysis due to either missing data or unengaged responses. These employees were working in lower- or middle-level management with designations such as business analyst, technical lead, product manager, subject matter expert, senior executive, executives, manager innovation, and associate manager. Out of these 454 respondents, 61.24% of the employees (*n* = 278) were males and 38.76% of the employees were females (*n* = 176). The average age of the respondents was 36.1 years, and the range of the age varies from 24 to 58 years. Furthermore, the average experience of the employees was 4.8 years with a standard deviation of 4.60 years. The majority of the respondents were married (71.15%), and 131 employees (28.85%) were unmarried.

The researchers selected IT and pharmaceutical companies as it is among the fastest growing sectors of India. It is expected that by 2020, India’s national policy related to IT aims to make India a global IT hub. Further, by 2020, it is expected that the pharmaceutical and health care sector will contribute US$ 55 billion as revenue^1^. These are the two sectors in India where the R&D share is high. There are many motives to select the R&D department for the present study. R&D competencies have emerged as one of the primary attributes that help organizations to differentiate on the basis of an organization’s performance (Teece, 1982; Bettis and Hitt, 1995; Nerkar and Paruchuri, 2005). The globalization of markets, the regionalization of scientific expertise, and the rapid change in technologies are forcing technology-oriented companies to continuously develop their R&D departments (Nixon, 1998; Gassman and Von Zedtwitz, 1999; Dushnitsky and Lenox, 2005; Mittal et al., 2019). In response to these challenges, modern organizations are appropriately developing R&D setups to enhance their skills that are essential for an organization’s success. Furthermore, leaders managing R&D teams face complex situations because of their dual responsibilities, i.e., firstly, they have to assist team members in developing their own competencies; secondly, they are also accountable for attaining results within several operational constraints (Frischer, 1993; de Weerd-Nederhof, 2000; Stoker et al., 2001; Pieterse et al., 2010). To understand the role of leaders in positively shaping an individual’s and organization’s performance, it becomes imperative to understand how leaders develop a relationship at various stages with their subordinates.

The selection of statistical tools and the characteristics of the research under consideration determine the adequate sample size for any research (Aggarwal et al., 2018a). Structural equation modeling (SEM) requires a relatively large sample size as SEM is sensitive to the magnitude of the sample (Schumacker and Lomax, 1996; Siddiqui, 2013). Therefore, we require a large sample size in the present study as we have used SEM for analyzing the proposed hypothesized relationships. Different authors have proposed different methods to determine the sample size (Aggarwal et al., 2018b). For example, some authors have proposed that the sample should be determined on the basis of distinct parameters in a model (Bentler and Chou, 1987; Bagozzi and Yi, 1988; Bollen, 1989; Hair et al., 1998; Kline, 1998). According to these, for each parameter, there must be at least five respondents. In the current study, there are 44 distinct parameters and we collected the data from 454 participants, which show that we have at least 10 respondents per parameter.

### Measures

#### Leader–Member Exchange

The subordinates were asked to rate their relationship with their immediate supervisor by using a seven-item questionnaire developed by Graen and Uhl-Bien (1995). These seven questions were asked on a five-point Likert scale anchoring from (1) strongly disagree to (5) strongly agree. The sample items consist of “Do you know where you stand with your leader? Do you usually know how satisfied your leader is with what you do? Regardless of how much formal authority he/she has built into his/her position, what are the chances that your leader would use his/her power to help you solve problems in your work? The value of the reliability coefficient for the LMX scale was 0.90.

#### Psychological Empowerment

The perceptions of psychological empowerment were measured with the scale developed by Spreitzer (1995). The scale consists of 12 items, and it is divided into four subscales, namely, competence, impact, self-determination, and meaning. Each subscale consists of three items which were measured on a five-point Likert scale. The sample items consist of “The work I do is very important to me (Meaning), I am self-assured about my capabilities to perform my work activities (Competence), I have considerable opportunity for independence and freedom in how I do my job (Self-Determination), I have a great deal of control over what happens in my department (Impact).” The results of the confirmatory factor analysis (CFA) supported a four-factor model of psychological empowerment such that χ^2^ = 92.138, *df* = 48, *p* > 0.001, χ^2^/*df* = 1.923 ≤ 3, root mean square residual (RMR) = 0.051 ≤ 0.08, root mean square error of approximation (RMSEA) = 0.045 ≤ 0.08, adjusted goodness of fit index (AGFI) = 0.946 ≥ 0.90, comparative fit index (CFI) = 0.987 ≥ 0.90, goodness of fit index (GFI) = 0.967 ≥ 0.80. Cronbach’s alpha of four dimensions of psychological empowerment was competence (0.869), impact (0.888), self-determination (0.818), and meaning (0.937). The overall scale reliability was 0.836, which is more than the cutoff value of 0.7.

#### Work Engagement

In order to measure the level of work engagement of the employees, a 17-item scale developed by Schaufeli et al. (2002) was applied. The scale is divided into three subscales, namely, vigor (six items), dedication (five items), and absorption (six items). The sample items consist of “When I get up in the morning, I feel like going to work (Vigor), I find the work that I do full of meaning and purpose (Dedication), When I am working, I forget everything else around me (Absorption).” IT was measured on a five-point Likert scale. An employee is said to have a high level of work engagement when he/she got a high score on these three dimensions. The results of the CFA supported a three-factor model of work engagement such that χ^2^ = 283.577, *df* = 114, *p* > 0.001, χ^2^/*df* = 2.488 ≤ 3, GFI = 0.932 ≥ 0.80, CFI = 0.959 ≥ 0.90, AGFI = 0.908 ≥ 0.90, RMSEA = 0.057 ≤ 0.08, RMR = 0.073 ≤ 0.08. The Cronbach’s alpha of three dimensions of work engagement was vigor (0.897), dedication (0.876), and absorption (0.874). The overall scale reliability was 0.861, which is more than the cutoff value of 0.7.

#### Psychological Withdrawal Behavior

The perceptions of the psychological withdrawal behavior of the employees were measured through the eight-item scale developed by Lehman and Simpson (1992). A five-point Likert scale was used to measure the intensity of agreement and disagreement of the respondents toward a particular statement. The sample item consists of “In the last 12 months, how often have you” “…Thoughts of being absent, Chat with coworkers about nonwork topics, Left work station for unnecessary reasons, Put less effort into job than should have.” The Cronbach’s alpha for this scale was 0.915.

## Data Analysis

Before analyzing the data, first, we performed a preliminary analysis for checking the suitability of the data. Then, we ran exploratory factor analysis to extract unrelated factors. After extracting the factors, we followed the two-step statistical analysis approach specified by Anderson and Gerbing (1988). In this, first, the CFA was performed to check the reliability and validity of the factors through the measurement model. Second, the proposed hypothesized relationships were tested using structural equation modeling.

### Preliminary Analysis

In the first step, with the help of Microsoft Excel, the data were checked for missing responses. The missing data were replaced with the arithmetic mean by following a simple imputation procedure (Byrne, 2010). The missing data were not an issue in the present study as they do not surpass 5% (Tabachnick and Fidell, 1996). In the second step, the data were checked for multivariate outliers using Mahalanobis Distance (D^2^) for each case (Byrne, 2010). There was no issue of multivariate outliers in the current study. The kurtosis and skewness were also checked to test the normality assumption, and the observed values do not exceed between +2 and -2 as recommended by Garson (2012).

### Common Method Bias

As the research design of the present study was cross-sectional and we collected the data from the respondents through the self-reported method, therefore, there might be an issue of common method bias (CMB) (Podsakoff et al., 2003). Therefore, in order to reduce the effect of CMB, firstly, the researchers selected observed variables in such a way that it incorporates reverse item questions which is an effective way of reducing CMB. Secondly, the researchers collected the data in such a way that at one point in time, only independent variables were measured (“Leader–Member Exchange”). With a gap of a fortnight, the researchers measured mediator (“Psychological Empowerment”) and dependent variables (“Work Engagement and Psychological Withdrawal Behavior”). When we gather the data in such a way, it potentially reduces the effect of CMB (Atwater and Carmeli, 2009). However, there still might be the effect of CMB in the data as we have collected the data at one point of time from the respondents in the case of the mediator and dependent variables. Therefore, to test it statistically, we performed Harman’s single-factor analysis (Shkoler and Tziner, 2017; Manohar et al., 2019). All the manifested variables were a constraint to unrotated one single factor using exploratory factor analysis (EFA) in SPSS 20.0 software. The single factor so generated exhibited a variance of 18.24%, which was lower than 50% total variance of the scale. This indicated the absence of CMB.

## Results

### Exploratory Factor Analysis

According to Cautin and Lilienfeld (2015), in order to have a scientifically justified outcome of CFA, a researcher should select the manifested variables in a measurement model based on the results of EFA. Therefore, EFA was applied on 44 statements by using the maximum likelihood extraction method based on eigenvalues greater than 1 (Henson and Roberts, 2006). In order to have distinct discrepancies among statements, we have selected maximum likelihood estimation. Further, the varimax method of orthogonal rotation was used to extract the factors. Prior to the extraction of factors, appropriateness of EFA was tested by assessing the values of Kaiser–Meyer–Olkin (KMO) and Bartlett’s test of sphericity. Results show that the value of KMO (0.864), which is more than the cutoff value of 0.6 (Kaiser and Rice, 1974), is significant at 0.01 level of the confidence interval. Further, the results of the EFA showed that all the variables have a standardized factor loading of more than 0.5 (Guadagnoli and Velicer, 1988). The results of EFA render 11 distinct factors that were labeled as “Leader–Member Exchange, Competence, Impact, Self-Determination, Meaning, Psychological Withdrawal Behavior, Vigor, Dedication, and Absorption.” Apart from this, none of the extracted factors explained substantially large variance. This is the indication that in the current study, there is no problem with CMB. Further, the results of total variance extracted showed that cumulatively, these 11 factors explain 70.15% of variance, which is more than the minimum acceptable critical value (Costello and Osborne, 2005).

The first factor was labeled as “Psychological Withdrawal Behavior.” It consists of eight items, and the reliability estimation of this construct came out to be 0.915. The second construct was labeled as “Leader–Member Exchange.” It consists of seven items, and the reliability estimation of this construct was 0.900. The third construct was labeled as “Vigor.” It consists of six items, and the reliability estimation of this construct was 0.897. The fourth construct was labeled as “Absorption.” It again consists of six items, and the value of reliability estimation of this construct was 0.874. The fifth construct was labeled as “Dedication.” It consists of five items, and the value of reliability estimation of this construct was 0.876. The sixth construct was labeled as “Meaning.” It consists of three items, and the reliability estimation of this construct was 0.937. The seventh construct was labeled as “Impact.” It consists of three items, and the value of reliability estimation of this construct was 0.888. The eighth construct was labeled as “Competence.” It consists of three items, and the reliability estimation of this construct was 0.869. The ninth construct was labeled as “Self-Determination.” It consists of three items, and the reliability estimation of this construct was 0.818. The results of the CFA were used to test the discriminant and convergent validity of the proposed hypothesized model.

### Confirmatory Factor Analysis

The reliability and construct validity of the latent variables were tested to confirm the adequacy of the measurement model. The construct validity of the model was measured through discriminant validity and convergent validity (Hair et al., 2006). In order to establish the discriminant validity, the model fit values of the hypothesized nine-factor model were compared with their competing conceptual model. The model fit value of the nine-factor model showed superior model fit values as compared to its competing models.

Results of Table 1 showed that the model fit value of hypothesized nine-factor model (Model 1) was significantly superior [χ^2^ = 1,405.45, *df* = 866, *p* > 0.001, χ^2^/*df* = 1.62 ≤ 3, RMSEA = 0.037 ≤ 0.08, p of close fit (Pclose) = 1.00; Standardized root mean square residual (SRMR) = 0.044 ≤ 0.08; non-normed fit index (NNFI) = 0.949 ≥ 0.90; CFI = 0.953 ≥ 0.90, GFI = 0.872 ≥ 0.80; expected cross-validation index (ECVI) = 3.65] than that of model 2 (Δχ^2^ from Model 1 = 1,613.40, *p* < 0.001). Results of Table 1 manifested that the model fit value of model 3, model 4, model 5, model 6, model 7, and in last model 8 showed poor model fit values as compared to model 1. Consequently, model 1 was retained for the final analysis with nine factors.

**TABLE 1:** Comparison of measurement models.

Model	Description	χ^2^	df	χ^2^/df	CFI	GFI	RMSEA	Δχ^2^ from Model 1	Δdf
Model 1	Hypothesized	1405.45	866	1.62	0.953	0.872	0.037	–	–
Model 2	Eight factor^a^	3018.85	874	3.45	0.813	0.687	0.074	1613.40***	8
Model 3	Seven factor^b^	4424.94	881	5.02	0.691	0.592	0.094	3019.49***	15
Model 4	Six factor^c^	5081.84	887	5.73	0.634	0.550	0.102	3676.39***	21
Model 5	Five factor^d^	6649.44	892	7.455	0.498	0.461	0.119	5243.99***	26
Model 6	Four factor^e^	5301.58	896	5.92	0.616	0.579	0.104	3896.13***	30
Model 7	Three factor^f^	6547.29	899	7.28	0.508	0.519	0.118	5141.84***	33
Model 8	Two factor^g^	8440.29	901	9.37	0.343	0.420	0.136	7034.84***	35

^a^Eight factor: psychological withdrawal behavior and leader-member exchange combined. ^b^Seven factor: psychological withdrawal behavior, leader-member exchange and vigor combined. ^c^Six factor: psychological withdrawal behavior, vigor, dedication and absorption combined. ^d^Five factor: psychological withdrawal behavior, leader-member exchange, vigor, dedication and absorption combined. ^e^Four factor: vigor, dedication and absorption combined and competence, impact, self-determination and meaning combined. ^f^Three factor: vigor, dedication, absorption, competence, impact, self-determination and meaning combined. ^g^Two factor: vigor, dedication, absorption, competence, impact, self-determination, meaning, psychological withdrawal behavior combined ***p < 0.001.

The convergent validity can be assessed by evaluating whether the standardized factor loadings of each statement are significant at its assigned factor or not (Anderson and Gerbing, 1988). The results of the measurement model showed that all the statements have standardized factor loading values above the specified criterion of 0.7 (Hair et al., 2010) and were also significant at *p* < 0.01 (Hair et al., 1998). Therefore, the present measurement model fulfills the conditions of convergent validity. In addition to this, convergent validity was assessed by the procedure specified by Hair et al. (2010), which states that the value of composite reliability (CR) of each factor should be greater than average variance extracted (AVE), and AVE should be greater than or equal to 0.5 (Fornell and Larcker, 1981). Results of the measurement model showed that all the factors have AVE more than 0.5, the value of CR for all the constructs is more than 0.7, and the value of CR is greater than AVE for each construct. This shows that the present measurement model has good convergent validity (Dhaliwal et al., 2019; Mittal et al., 2020). Further, the discriminant validity was checked by two methods. The first method states that the correlation values among the factors should be below the cutoff value of 0.85 (Kline, 2015). The second method states that the value of the square root of AVE for each factor should exceed the value of the correlation of that construct with other constructs in the model (Fornell and Larcker, 1981). The results of the measurement model fulfill both of these criteria, thereby proving the evidence of discriminant validity in the measurement model (Table 2). The results of Table 2 revealed that all the constructs have a high internal consistency as the value of CR estimation for all the constructs is higher than 0.7 (Nunnally, 1978).

**TABLE 2:** Reliability and validity of the measurement model.

	CR	AVE	1	2	3	4	5	6	7	8	9
Absorption	0.88	0.54	**0.74**								
LMX	0.90	0.56	0.2	**0.75**							
Competence	0.87	0.69	0.13	0.14	**0.83**						
Impact	0.88	0.73	0.3	0.2	0.26	**0.85**					
Self	0.82	0.61	0.11	0.11	0.13	0.2	**0.78**				
Meaning	0.94	0.84	0.34	0.28	0.32	0.57	0.17	**0.92**			
PWB	0.92	0.57	–0.07	–0.14	–0.12	–0.09	–0.01	–0.03	**0.76**		
Vigor	0.89	0.59	0.27	0.13	0.16	0.17	0.11	0.19	–0.18	**0.77**	
Dedication	0.88	0.59	0.25	0.23	0.09	0.14	0.07	0.28	–0.36	0.25	**0.77**

*1(Absorption); 2(Leader-Member Exchange); 3(Competence); 4(Impact); 5(Self-Determination); 6(Meaning); 7(Psychological Withdrawal Behaviour); 8(Vigor); 9(Dedication). Bold diagonal values represent square root of respective AVE and off-diagonal values are inter-construct correlation. CR represent Composite Reliability.*

### Descriptive and Correlation Analysis

The results of Table 3 depicted the descriptive, correlation, and reliability coefficients. Results showed that there is a significant negative relationship between LMX and psychological withdrawal behavior (*r* = −0.745, *p* < 0.01). It means that if an employee falls under the in-group category, then the withdrawal behavior among the employee reduces. Results showed that there is a significant positive relationship between LMX and psychological empowerment (*r* = 0.504, *p* < 0.01).

**TABLE 3:** Descriptive statistics, correlation and reliability.

Factors	Mean	*SD*	1	2	3	4
Leader-member exchange	3.016	0.703	**(0.900)**			
Psychological withdrawal behavior	2.873	0.99	−0.745**	**(0.915)**		
Psychological empowerment	4.054	0.893	0.504**	−0.545**	**(0.859)**	
Work engagement	4.032	0.844	0.682**	−0.737**	0.462**	**(0.861)**

Off-diagonal values are inter-construct correlation; **p < 0.01. Bold values represent reliability coefficients of the corresponding construct.

In addition to this, results showed that LMX has a significant and positive relationship with work engagement (*r* = 0.682, *p* < 0.01). It means that a high-quality relationship between leader and members has a positive impact on employees’ work engagement. Psychological withdrawal behavior has a significant and negative relationship with psychological empowerment (*r* = −0.545, *p* < 0.01), such that high perceptions of psychological empowerment will lead to low levels of psychological withdrawal behavior among the employees. A similar type of result was observed in case of psychological withdrawal behavior and work engagement. Results showed a significant and negative relationship between psychological withdrawal behavior and work engagement (*r* = −0.737, *p* < 0.01), and the strength of the relationship is also strong. It means that as the work engagement among the employees increases, there is a reduction in the withdrawal behavior among employees. Finally, the results of the correlation analysis revealed that there is a significant positive relationship between psychological empowerment and work engagement (*r* = 0.462, *p* < 0.01). It means that as the employee feels empowered at his/her workplace, his/her engagement toward work increases.

### Structural Model

**FIGURE 1:**
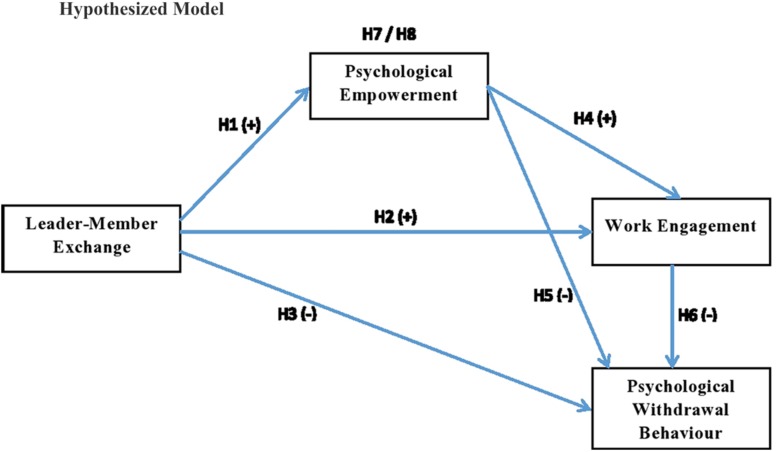
Hypothesized model.

In the present study, researchers have used the structural equation modeling technique to test the hypothesized relationships between psychological empowerment, LMX, work engagement, and psychological withdrawal behavior (see Figure 1). The benefit of using structural equation modeling technique is that it allows multi-construct variables to be treated as one single latent variable and use the scale means of its sub-facets as its measurement indicators in the path analysis (Aryee and Chen, 2006; Iacobucci, 2008; Schermuly et al., 2013). Therefore, in the present study, the loadings of the four sub-factors of psychological empowerment (meaning, competence, self-determination, and impact) and three subdimensions of work engagement (vigor, dedication, and absorption”) capture the gestalt of psychological empowerment (Spreitzer, 1995) and work engagement (Schaufeli et al., 2002) in the context of our sample.

The researchers have also controlled certain demographical variables such as gender, educational qualification, age, marital status, and length of service (Aggarwal et al., 2019a). The critical ratio values rendered by regression estimates were utilized to test the relationship between two variables (Biswas et al., 2006). A critical ratio (*t*-value) ≥ 1.96 but smaller than 2.58 indicates that the relationship between two variables is significant at 95% confidence interval, whereas if the value of critical ratio is greater than 2.58, then it means that the relationship between two variables is significant at 99% of the confidence interval.

In order to test the problem of endogeneity, we run the proposed structural model in reverse order. The results of the model fit of this reversed model depicts the value of χ^2^ = 304.883, *df* = 14, *p* > 0.001, χ^2^/*df* = 21.777; RMSEA = 0.214, Pclose = 0.00; SRMR = 0.1314; NNFI = 0.679 ≥ 0.90; CFI = 0.840; GFI = 0.866. The results of the model fit shows that the proposed structural model is having a better model fit than a reversed model (χ^2^ = 39.9, *df* = 15, *p* > 0.001, χ^2^/*df* = 2.66; RMSEA = 0.042, Pclose = 1.00; SRMR = 0.051; NNFI = 0.923 ≥ 0.90; CFI = 0.934; GFI = 0.896).

The results of Table 4 showed that there is a significant positive effect of LMX on the perceptions of the psychological empowerment of the employees (β = 0.504, critical ratio = 12.431). Hence, H1 was accepted. Results of the path analysis also showed that there is a significant positive impact of LMX on work engagement (β = 0.599, critical ratio = 15.387). Therefore, H2 was accepted. In addition to this, results of Table 4 showed that the quality of LMX has a negative impact on the psychological withdrawal behavior of the employees (β = −0.628, critical ratio = −18.180), and H3 was accepted. The results of Table 4 showed that psychological empowerment has a positive impact on the work engagement of the employees (β = 0.165, critical ratio = 4.242). Hence, H4 was accepted. The results of Table 4 showed that there is a significant and negative relationship between psychological empowerment and psychological withdrawal behavior (β = −0.230, critical ratio = −6.658). Therefore, H5 was accepted. Last, the results of the structural model showed that work engagement has a negative impact on employees’ psychological withdrawal behavior (β = −0.776, critical ratio = −39.050). Therefore, H6 was accepted.

**TABLE 4:** SEM standardized coefficients.

	Relationship	Std β	*t*-value	Decision
H1	Leader-member exchange → Psychological empowerment	0.504	12.431***	Accepted
H2	Leader-member exchange → Work engagement	0.599	15.387***	Accepted
H3	Leader-member exchange → Psychological withdrawal behavior	–0.628	−18.180***	Accepted
H4	Psychological empowerment → Work engagement	0.165	4.242***	Accepted
H5	Psychological empowerment → Psychological withdrawal behavior	–0.230	−6.658***	Accepted
H6	Work engagement → Psychological withdrawal behavior	–0.776	−39.050***	Accepted

***p < 0.01.

In the present study, the bootstrapping technique with a bias-corrected confidence interval at 95% confidence level with resampling at 2,000 was used to examine the mediating role of psychological empowerment on the proposed relationships. This method states that the standardized indirect effect is considered statistically significant when bias-corrected confidence interval (lower bound and upper bound) does not contain zero (Shrout and Bolger, 2002; MacKinnon et al., 2007). The results of Table 5 show that psychological empowerment mediates the relationship between LMX and work engagement. In addition to this, the results of the bootstrapping analysis revealed that psychological empowerment mediates the relationship between LMX and psychological withdrawal behavior. Therefore, in order to have a harmonious relationship in the working environment, employers should focus on activities that enhance the feeling of psychological empowerment among the employees.

**TABLE 5:** SEM bootstrapping confidence intervals (95% CI, 2,000 resamples).

	Relationship	Direct effect	*p*-value	Indirect effect	*p*-value	Mediation
H7	Leader-member exchange → Psychological empowerment → work engagement	0.599	0.001	0.083	0.002	Partial
H8	Leader-member exchange → Psychological empowerment → Psychological withdrawal behavior	-0.628	0.001	-0.116	0.002	Partial

*Source: Author’s Compilation.*

## Discussion

Results of the study manifested that LMX positively impacts psychological empowerment such that employees with high dyadic relationships perceive a high level of psychological empowerment, whereas employees with low dyadic relationships perceive a low level of psychological empowerment. It means that employees who perceive that they have a high-quality relationship with their leader/manager perceive high levels of competence, impact, self-determination, and meaning (Liden et al., 2000; Gomez and Rosen, 2001; Aryee and Chen, 2006; Laschinger et al., 2007; Harris et al., 2009; Zhong et al., 2011; Hill et al., 2014; Schermuly and Meyer, 2016). In addition to this, results showed that LMX affects work engagement positively. It means that in-group members showed a high level of work engagement as compared to out-group members. The finding of the present study is in line with prior research (Li et al., 2012; Burch and Guarana, 2014; Breevaart et al., 2015; Gutermann et al., 2017). Further, results showed that LMX affects the employees’ withdrawal behavior in a negative manner such that for employees who are part of the in-group, their perceptions toward psychological withdrawal behavior are less as compared to out-group members. The cause for such type of relationship might be because employees with high dyadic relationships receive a high level of benefits, emotional support, and trust as compared to employees who are not part of the in-group (Dansereau et al., 1975; Dienesch and Liden, 1986). On the contrary, out-group members develop negative feelings toward their leaders. As their leader gives fewer benefits, they have less communication and show less amount of trust in out-group members (Schneider, 1987). Therefore, out-group members develop negative feelings toward their leaders. As the relationships between leader and out-group members are not congenial, therefore the members of the out-group will try to avoid this relationship either by reducing the interaction with the leader or by withdrawing from the job. Results of the path analysis showed that psychological empowerment positively impacts employees’ work engagement, such that when an employee perceives the high level of psychological empowerment at the workplace, his/her engagement toward the work will be high. On the contrary, if an employee perceives a low level of psychological empowerment, in that case, the work engagement of the employee will be less. The results of the present study are in line with prior research which states a positive relationship between psychological empowerment and work engagement (Stander and Rothmann, 2010; De Villiers and Stander, 2011; Bhatnagar, 2012; Ugwu et al., 2014; Moura et al., 2015). Previous research found that employees were highly engaged when they perceive psychological safety and meaningfulness at their workplace (Kahn, 1990; Saks, 2008). Similarly, Cho et al. (2006) stated that an empowered employee mostly found meaning at the workplace and at the same time he/she is highly motivated. Further, this motivation at the workplace helps the empowered employee to achieve organizational effectiveness by working at his/her goals which are related to the job (Kanter, 1979). On the contrary, when an employee does not feel empowered at the workplace, then it results in a low level of commitment, less employee engagement, intrinsic motivation, job satisfaction, high level of turnover intentions, withdrawal behavior, and burnout (Cho et al., 2006; De Villiers and Stander, 2011; Bhatnagar, 2012; Lee, 2015; Moura et al., 2015; Wang and Liu, 2015; Aggarwal et al., 2018a). Zhang and Bartol (2010) suggested that empowering leadership in the workplace will result in a creative, intrinsic, motivated, and engaged employee. Therefore, an employer should focus on empowering the employees at the workplace as it has various positive and negative consequences that affect both employee and organizational performance. The result of the path analysis showed that there is a negative relationship between psychological empowerment and employees’ psychological withdrawal behavior, such that employees with high perceptions of psychological empowerment at the workplace will have fewer chances of withdrawal behavior as compared to employees who perceive a low level of psychological empowerment. The finding of the present study is in line with previous empirical work (Shapira-Lishchinsky and Rosenblatt, 2010; Shapira-Lishchinsky and Tsemach, 2014). Seibert et al. (2011) found that high perceptions of psychological empowerment have a negative impact on the turnover intention of the employees. Erdogan and Bauer (2009) conducted a study on 244 sales associates working in 25 Turkish retail stores. The results of the study showed that psychological empowerment was negatively associated with voluntary turnover and intention to leave. A similar type of result was replicated in Fook et al. (2011) study, which showed a negative association between psychological empowerment and employees’ withdrawal intentions. Negative attitudes toward work such as intention to leave, spending work time on personal matters, intentionally reducing the work efforts, voluntary absenteeism, and lateness are all subdimensions of psychological withdrawal behavior (Koslowsky, 2009; Shapira-Lishchinsky and Rosenblatt, 2010; Biron and Bamberger, 2012; Erdemli, 2015). Therefore, it is important to study those factors that affect psychological withdrawal behavior so that corrective actions could be taken.

## Implications

The present study contributes extensively to the area of psychological withdrawal behavior as the authors were not able to find a single study that examines all the four variables (LMX, psychological empowerment, work engagement, and psychological withdrawal behavior) in one study that too in research and development context. Results of the present study postulated that the relationship between subordinates and supervisor plays a vital role in affecting organizational and individual-level outcomes such as perceived organizational support (Kath et al., 2010), organizational citizenship behavior (Kim et al., 2010; Sun et al., 2013; Aggarwal and Singh, 2016; Singh et al., 2020), organizational commitment (Lo et al., 2010; Le Blanc and González-Romá, 2012; Saeed et al., 2014), psychological empowerment (Aryee and Chen, 2006; Laschinger et al., 2007; Hill et al., 2014; Schermuly and Meyer, 2016), job satisfaction (Loi et al., 2014; Li et al., 2018), work engagement (Li et al., 2012; Runhaar et al., 2013; Breevaart et al., 2015), and turnover intentions (Harris et al., 2014; Li et al., 2018). According to the LMX theory, relationships are built over time through positive exchanges that produce loyalty, mutual respect, and high performance (Graen and Uhl-Bien, 1995; Liden et al., 2006). Therefore, leaders must pay the utmost attention to maintain a harmonious relationship with their employees. However, in this present competitive business environment where supervisors have generally large spans of control, it becomes difficult for the supervisors to have a harmonious and high-quality relationship with each and every member. This results in a jeopardized situation for a manager, where he/she has fewer opportunities to interact with his/her subordinates and fewer chances of reciprocating to the efforts of a subordinate. Therefore, the results of the present study can help a manager in reducing the negative feelings among employees and enhance the positive feelings related to work and organization. The results of the present study are consistent with the past studies which state a positive relationship between high-dyadic relationships and psychological empowerment (Brunetto et al., 2012; Schermuly and Meyer, 2016; Srivastava and Dhar, 2016). When an employee feels highly empowered in terms of meaning at his/her workplace, then the employee feels more confident in his/her capabilities and try to achieve the self-actualization level (Gerstner and Day, 1997; Gomez and Rosen, 2001). In a high-quality LMX relationship, generally, there is a sense of mutual trust and respect among leaders and members. The leader in return enhances the empowering working conditions for them such as giving scare resources, flexibility in decision making, etc. This suggests that a positive working relationship is necessary to optimize the value of these empowering strategies for managers. This high dyadic and empowering situation at the workplace further results in high work engagement and low psychological withdrawal behavior. The present study also contributes to the existing literature as this study examines the work engagement and psychological empowerment as a multilevel framework and how the quality of a dyadic relationship affects these two dependent variables. Further, the results of the present study revealed that psychological empowerment mediates the relationship between LMX and work engagement. It means that the type of leadership style experienced by an empowered subordinate will lead to more control at the workplace and enhance his/her intrinsic motivation, which further resulted in a high level of engagement by the subordinate. As an intrinsic source of motivation, the experience of empowerment enhances levels of job satisfaction and work engagement and reduces psychological withdrawal behavior. In addition to this, the results of the present study showed that psychological empowerment mediates the relationship between LMX and psychological withdrawal behavior. It means that when an employee has a high-quality relationship with his/her supervisor and he/she feels empowered at the workplace, then the intensity of psychological withdrawal behavior will be less. Therefore, a manager must pay utmost attention to developing high-quality relationships with most of the employees, and the policies of the management must be in such a way that they empower the employees. As India is a society with both individualistic and collectivistic traits, therefore, the findings of the present study can be generalized to other countries.

## Limitations

Although the current study has given valuable information pertaining to the variables under consideration, still, there are some limitations of this study, and we need to take care of these limitations while generalizing the results of the current study. The first limitation is related to the way of collecting the data. As in the current study, the data were collected through the self-reported method. Therefore, there might be an issue of CMB. In order to handle this limitation, we have collected the data in two phases. At the first point of time, we collected the data for the independent variables, and at the second point of time, we collected the data for the mediator and dependent variables. Apart from this, the CMB is not a major concern in those studies which make use of well-designed multi-factor statements (Spector, 1987). Although researchers tried their best to minimize the effect of CMB, each remedy has its own disadvantages (Podsakoff et al., 2003). The second limitation is related to the use of a quantitative method of analyzing the data. Future researchers could use both quantitative and qualitative analyses to get more insight into the current topic. Third, there might be some other important variables (mediator/independent variables) that would have affected the psychological withdrawal behavior. Future researchers could expand the current model by taking more variables under study. Fourth, the current study has taken psychological empowerment and work engagement in the second order. In prior literature, the majority of the researchers have taken these variables in the second order. The future researchers could take these variables as a multidimensional construct in order to have a deeper understanding of the relationship. As the results of the present research are based on the responses given by respondents through the self-reported method, there might be a chance that they might have given socially acceptable answers. Therefore, this raises a concern for the validity of the questionnaire used in the present research (Bennett and Robinson, 2000). Further, the present research was conducted at one point in time. Therefore, in order to have more concrete results, a longitudinal study is required especially in terms of LMX as it is dynamic in nature. In addition to this, the current research has used a shorter version of LMX, although well established in prior literature, this might lead to biases in the results at large. Future researchers could use a fuller version of the variables used in the present research.

## Conclusion

This study tried to explore the psychological withdrawal behavior model in R&D employees and pinpoints the importance of the behavioral and organizational factors affecting the behavior of the employees working in public and private sectors. The findings of the current study proposed that the organizations must reconsider and revise their existing policies related to employees in such a way that they empower the employees and give a fair chance to develop a good interpersonal relationship not only with peer groups but also with their immediate supervisor as the quality of the relationship with the supervisor has severe consequences at the individual and organizational levels. Finally, the current study uses a rigorous methodology in collecting the data and used SEM to analyze the data. Therefore, the results of the present study are accurate and reliable, which can be further generalized to a large extent.

## Data Availability Statement

The datasets for this article are not publicly available because the data is still being used for additional research. Requests to access the datasets should be directed to the corresponding author.

## Ethics Statement

Ethical review and approval was not required for the study on human participants in accordance with the local legislation and institutional requirements. Written informed consent for participation was not required for this study in accordance with the national legislation and the institutional requirements.

## Author Contributions

AA has conceptualized the topic, written the Research Methodology section, done the formal data analysis, and interpretation part of the data analysis section. PC has written the theoretical framework and hypotheses development of the manuscript. DJ has written the Introduction section. AM has written the Implications, Limitations, and Conclusion sections.

## Conflict of Interest

The authors declare that the research was conducted in the absence of any commercial or financial relationships that could be construed as a potential conflict of interest.

## Footnotes

1 https://www.ibef.org/industry/research-development-india.aspx
